# Genetic Identification of a PilT Motor in *Geobacter sulfurreducens* Reveals a Role for Pilus Retraction in Extracellular Electron Transfer

**DOI:** 10.3389/fmicb.2016.01578

**Published:** 2016-10-17

**Authors:** Allison M. Speers, Bryan D. Schindler, Jihwan Hwang, Aycin Genc, Gemma Reguera

**Affiliations:** Department of Microbiology and Molecular Genetics, Michigan State UniversityEast Lansing, MI, USA

**Keywords:** type IV pili, pilus retraction, metal reduction, electroactive biofilms, pilus nanowires

## Abstract

The metal-reducing bacterium *Geobacter sulfurreducens* requires the expression of conductive pili to reduce iron oxides and to wire electroactive biofilms, but the role of pilus retraction in these functions has remained elusive. Here we show that of the four PilT proteins encoded in the genome of *G. sulfurreducens*, PilT3 powered pilus retraction in planktonic cells of a PilT-deficient strain of *P. aeruginosa* and restored the dense mutant biofilms to wild-type levels. Furthermore, PilT3 and PilT4 rescued the twitching motility defect of the PilT-deficient mutant. However, PilT4 was the only paralog whose inactivation in *G. sulfurreducens* lead to phenotypes associated with the hyperpiliation of non-retractile mutants such as enhanced adhesion and biofilm-forming abilities. In addition, PilT4 was required to reduce iron oxides. Taken together, the results indicate that PilT4 is the motor ATPase of *G. sulfurreducens* pili and reveal a previously unrecognized role for pilus retraction in extracellular electron transfer, a strategy that confers on *Geobacter* spp. an adaptive advantage for metal reduction in the natural environment.

## Introduction

The hallmark of the physiology of bacteria in the family *Geobacteraceae* is their ability to conserve energy for growth by transferring respiratory electrons to extracellular Fe(III) oxide minerals, a process that requires the expression of conductive type IV pili (T4P) (Reguera et al., 2005). *Geobacter* T4P are homopolymers of a single pilin subunit (PilA) (Cologgi et al., 2011), a short peptide (61 amino acids in the model representative *Geobacter sulfurreducens*) that retains the conserved amino-terminal features of type IVa pilins but diverges in amino acid composition and structure at the carboxy-terminus (Feliciano et al., 2012). This also places the *Geobacter* pilins in an independent line of descent among bacterial type IV pilins and pseudopilins (Reguera et al., 2005). A structural model of the pilus fiber optimized via molecular dynamics revealed the clustering of aromatic amino acids of the pilins once assembled and the formation of axial and transversal pathways with inter-aromatic distances and geometries optimal for multistep charge hopping (Feliciano et al., 2015). Consistent with this mechanism for charge transport, *in vitro* conductivity measurements of pili purified from *G. sulfurreducens* demonstrated the thermal dependence of incoherent conductivity (Lampa-Pastirk et al., 2016). Molecular dynamics simulations also revealed that some aromatic side chains can transiently get close to each other (3.5–5 Å), forming aromatic contacts that promote charge transport at rates (about one billion electrons per second along a 1 μm-long pilus at 100 mV) that greatly exceed the respiratory rates of the cell (Lampa-Pastirk et al., 2016). Indeed, an alanine replacement of one of the tyrosines (Y27) involved in the formation of half of the aromatic contacts in the pilus fiber (Feliciano et al., 2015) reduced the carrier mobility, five-fold (Lampa-Pastirk et al., 2016). Similarly, alanine replacements of the pilin's three tyrosines (Tyr3 mutant) reduced the number of aromatic contacts in half (Feliciano et al., 2015) and increased the electrical resistance of the T4P five-fold (Steidl et al., 2016). As a result, a Tyr3 mutant had growth defects in cultures with Fe(III) oxides, with generation times increasing four-fold compared to the wild-type strain (Feliciano et al., 2015).

In addition to their role as electronic conduits between the cell and Fe(III) oxides (Reguera et al., 2005) and uranium (Cologgi et al., 2011), the T4P of *G sulfurreducens* promote cell-cell aggregation and biofilm formation on various surfaces (Reguera et al., 2006, 2007; Cologgi et al., 2014; Steidl et al., 2016). T4P assembly and conductivity are also required for maximum current generation and electroactivity of anode biofilms grown in microbial electrolysis cells (MECs), which are devices operated with the anode electrode poised at a metabolically oxidizing potential to provide an unlimited electron acceptor for biofilm growth (Steidl et al., 2016). In electroactive biofilms, the T4P work coordinately with matrix-associated *c*-type cytochromes to transport charges to the underlying electrode until the biofilms grow to a threshold thickness (~10 μm) that limits the rates of electron transfer via the cytochromes (Steidl et al., 2016). The conductive T4P therefore become the primary mechanism to discharge respiratory electrons from cells positioned in the otherwise electron acceptor-limited outer regions of thick biofilms (Steidl et al., 2016).

In contrast to the number of mechanistic studies of pilus conductance, little is known about the components of the *Geobacter* T4P apparatus and their role in metal respiration and the formation of electroactive biofilms. The expression of T4P is a dynamic process controlled by antagonistic cycles of extension and retraction of the pilus fibers, which are powered by the PilB and PilT ATPases of the pilus apparatus, respectively (Jakovljevic et al., 2008). The genome of *G. sulfurreducens* contains a *pilB* and a *pilT* (*pilT4*) homolog in the same *pil* gene cluster where the pilin-encoding gene, *pilA*, is located (Reguera et al., 2005). The genetic arrangement of this region is similar to the *pil* region of *Myxococcus xanthus* (Wall and Kaiser, 1999), another delta-Proteobacterium, and also includes *pilC*, the gene encoding the PilC protein that couples the coordinated action of PilB and PilT in other bacteria (Takhar et al., 2013). This makes *pilB* and *pilT4* the most obvious candidates to encode for the T4P extension and retraction ATPases in *G. sulfurreducens*, respectively. Consistent with this prediction, deleting the *pilB* gene in *G. sulfurreducens* prevented pili assembly and the growth of biofilms on anode electrodes beyond the threshold thickness (~10 μm) that limits the rates of electron transfer via matrix-associated *c*-type cytochromes (Steidl et al., 2016). Furthermore, the electrochemical activity of the *pilB* biofilms was approximately half of that of wild-type anode biofilms grown to the same thickness, consistent with the requirement to produce pili for optimal electron transport to the underlying electrode (Steidl et al., 2016). The role of *pilT4* in pilus retraction in *G. sulfurreducens* remains however unclear, based on the lack of a measurable phenotype of a *pilT4* deletion mutant during the reduction of Fe(III) oxides (Reguera et al., 2005). Furthermore, twitching motility in *G. sulfurreducens*, which requires the retractile properties of the pili, has never been observed using submerged assays with glass coverslips with or without iron coatings (Reguera et al., 2005). Hence, the physiological relevance of pilus retraction in this organism remains uncertain.

Interestingly, the genome of *G. sulfurreducens* contains three additional *pilT* genes outside of the *pil* gene cluster (designated *pilT1, pilT2*, and *pilT3*), which could encode for a functional pilus retraction protein. The expression of one or more of these additional PilT proteins could have suppressed any phenotypes associated with the *pilT4* mutant in cultures growing with Fe(III) oxides (Reguera et al., 2005), similarly to the compensatory effects that are often observed when components of the respiratory machinery of *G. sulfurreducens* are inactivated (Kim et al., 2005, 2006, 2008; Leang et al., 2005; Cologgi et al., 2011, 2014; Steidl et al., 2016). It is also possible that *Geobacter* pili rely on the coordinated action of more than one PilT protein to power pilus retraction under specific conditions. PilU, for example, a well-characterized PilT paralog, is essential for PilT-mediated twitching motility in *Pseudomonas aeruginosa* (Whitchurch and Mattick, 1994). However, it is dispensable for pilus retraction (Park et al., 2002; Maier et al., 2004) and twitching motility (Maier et al., 2002; Park et al., 2002) in *Neisseria gonorrhoeae*, which are pili functions that require the activity of a third PilT paralog (PilT2) (Kurre et al., 2012). Further, PilT mediates T4P retraction at low velocity and high force in *M. xanthus*, but the pili also retract in a PilT-independent mode characterized by its high-velocity and low force, which is presumably powered by one or more of the other four PilT paralogs encoded by genes outside of the *pil* cluster (Clausen et al., 2009). Hence, the coordinated activity of more than one PilT ATPase may be required for specific PilT-mediated functions in *G. sulfurreducens*.

In this study, we took a genetic approach to identify functional PilT motors in *G. sulfurreducens* and, by extension, to investigate if pilus retraction is physiologically relevant in current-harvesting biofilms or during the reduction of Fe(III) oxides. We first expressed each of the *pilT* genes of *G. sulfurreducens* in the well-characterized PilT null mutant of *P. aeruginosa* strain K (Sundin et al., 2002) to assess the ability of the PilT proteins to complement functions in this organism that require PilT-mediated retraction. PilT3 and PilT4 were able to restore all or some of the defects of the pilus retraction deficiency in the heterologous host, respectively. However, PilT4 was the only paralog whose inactivation in *G. sulfurreducens* resulted in phenotypes expected for a retraction-defective mutant such as increased adhesion and formation of denser biofilms. Furthermore, PilT4 was also required for optimal electron discharge per cell in electroactive biofilms and during the reduction of Fe(III) oxides. The genetic data thus indicates that PilT4 is the main T4P retraction ATPase motor in *G. sulfurreducens* and supports a previously unrecognized role for PilT4-mediated pilus retraction in the formation and electroactivity of biofilms and during the respiration of Fe(III) oxides. We also discuss the possibility that other PilT paralogs, particularly PilT3, may be involved in fine-tuning pilus dynamics in *G. sulfurreducens* and the implications that these findings have on the environmental survival of these bacteria.

## Materials and methods

### Bacterial strains and growth conditions

All the strains of *P. aeruginosa* used in the heterologous expression experiments (Table 1) and *Escherichia coli* strains used for cloning and transformation were routinely grown aerobically in Luria-Bertani (LB) medium and incubated at 37°C with gentle agitation. *G. sulfurreducens* strain PCA was from our culture collection and was used as the wild-type background to construct all of the mutant derivatives (Table 1). All of the *Geobacter* strains were routinely cultured anaerobically in NB medium (Coppi et al., 2001) supplemented with 0.1% yeast extract and 1 mM cysteine and with 15 mM acetate and 40 mM fumarate (NBAF). When indicated, NBAF was replaced by a modified freshwater FW medium (Cologgi et al., 2011) supplemented with 1 μM Na_2_SeO_4_ to stimulate growth (Speers and Reguera, 2012) and with 15 mM acetate as electron donor and 40 mM fumarate as the electron acceptor (FWAF). Fe(III) oxides cultures contained FWA medium and 100 mM poorly crystalline Fe(III) oxides and, when indicated, 4 mM of the metal chelator nitrilotriacetic acid (NTA). Growth in the Fe(III) oxides cultures was measured periodically with the ferrozine method (Stookey, 1970) as the amount of 0.5 N HCl-extractable Fe(II) resulting from the reduction of Fe(III) (Lovley and Phillips, 1986). Unless otherwise indicated, all of the incubations were at 30°C.

**Table 1:** **Strains and plasmids used**.

**Strains and plasmids**	**Relevant characteristics^a^**	**Source or reference**
***P. aeruginosa***		
PAK	Wild type; piliated; twitching	Takeya and Amako, 1966
PAKΔ*pilA*	Δ*pilA*; non-piliated; non-twitching	Kagami et al., 1998
PAKΔ*pilT*	Δ*pilT*; hyperpiliated; non-twitching	Sundin et al., 2002
PAKΔ*pilT*-p	Δ*pilT* with empty pMMB67EH vector	This study
PAK*pilT1*	PAKΔ*pilT* complemented with pMMB-*pilT1*	This study
PAK*pilT2*	PAKΔ*pilT* complemented with pMMB-*pilT2*	This study
PAK*pilT3*	PAKΔ*pilT* complemented with pMMB-*pilT3*	This study
PAK*pilT4*	PAKΔ*pilT* complemented with pMMB-*pilT4*	This study
***G. sulfurreducens***		
WT	Wild type strain PCA	Caccavo et al., 1994
*pilT1*	*ΔpilT1*::*aaaC1*, Gm^r^	This study
*pilT2*	*ΔpilT2*::*aaaC1*, Gm^r^	This study
*pilT3*	*ΔpilT3*::*aaaC1*, Gm^r^	This study
*pilT4*	*ΔpilT4*::*aaaC1*, Gm^r^	This study
*ΔpilT4*	*ΔpilT4*::*loxP*	This study
*pilT4*p	*ΔpilT4* with empty pRG5 vector	This study
*pilT4*+	*ΔpilT4* complemented with pRG5-*pilT4*	This study
*pilT3 pilT4*	*ΔpilT4* strain carrying the *ΔpilT3*::*aaaC1* mutation, Gm^r^	This study
*pilB*	*ΔpilB*::*aaaC1*, Gm^r^	This study
*ΔpilB*	*ΔpilB*::*loxP*	This study
*pilB*+	*ΔpilB* complemented with pRG5-*pilB*	This study
*pilA*	*ΔpilA::cat*, Cm^r^	Reguera et al., 2005
**PLASMIDS**		
pCM351	Amp^r^, Tet^r^, Gm^r^, ColE1 *ori, oriT*	Marx and Lidstrom, 2002
pCM158	Km^r^, *trfA, oriT, oriV*, ColE1 *ori, cre*	Marx and Lidstrom, 2002
pCR2.1-TOPO	Amp^r^, Km^r^, ColE1 *ori*	Invitrogen
pRG5	*G. sulfurreducens* shuttle vector; Spc^r^; P*_*taclac*_*	Kim et al., 2005
pRG5-*pilT4*	pRG5 with *G. sulfurreducens pilT4*	This study
pRG5-*pilB*	pRG5 with *G. sulfurreducens pilB*	This study
pRK2013	Km^R^, ColE1 *ori, tra*1 (RK2), helper plasmid for PAK conjugation	Furste et al., 1986
pMMB67EH	PAK shuttle vector; Amp^R^, Mob+, P*_*taclac*_*	Furste et al., 1986
pMMB-*pilT1*	pMMB67EH with *G. sulfurreducens pilT1*	This study
pMMB-*pilT2*	pMMB67EH with *G. sulfurreducens pilT2*	This study
pMMB-*pilT3*	pMMB67EH with *G. sulfurreducens pilT3*	This study
pMMB-*pilT4*	pMMB67EH with *G. sulfurreducens pilT4*	This study
pMMB-*pilB*	pMMB67EH, *G. sulfurreducens pilB*	This study

### Quantitive real time PCR (qRT-PCR)

The WT strain of *G. sulfurreducens* was grown in 10 ml of NBAF or FWAF and cultures were incubated either at 30 or 25°C. Exponentially growing cells were then harvested by centrifugation, and the pelleted cells were suspended in 2 ml of TRIzol® reagent (Invitrogen Life Technologies). Extraction of total cellular RNA was performed following manufacturer's recommendations. Genomic DNA was removed with RNase-free DNase I (QIAGEN) and the remaining RNA was purified using a QIAGEN's RNeasy column. Primers were designed to have the same melting temperature and PCR efficiency using the pcrEfficiency open source tool (http://srvgen.upct.es/efficiency.html; Mallona et al., 2011). Complementary strand synthesis was performed in a 20 μl reaction mixture containing 1 μg of total RNA, 10 pmol of gene-specific primer and dNTPs. The mixture was heat-treated at 65°C for 5 min followed by incubation on ice for at least 1 min before adding 1 μl of SuperScript® III reverse transcriptase (Invitrogen) and incubating at 50°C for 60 min. The reaction was inactivated by heating at 70°C for 15 min. PCR-amplification reactions (50 μl, total volume) contained 1 μl of cDNA (serial dilutions from first-strand synthesis reaction), 10 pmol of sense and antisense primers (Table 2), and 25 μl of SYBR green 2X master mix (Applied Biosystems, Life Technologies). qRT-PCR was performed in duplicate reactions under conditions (95°C for 30 s, 50°C for 30 s, and 50 cycles of 1 min at 70°C) that resulted in threshold crossings between 18 and 35 for all of the primer pairs. Fold changes in gene expression of the target genes were calculated in reference to the internal control *recA* as $2^{-\Delta {\text{C}}_{\text{T}}}$ (Schmittgen and Livak, 2008), where ΔC_T_ is the C_T_ of the gene of interest minus the C_T_ of the *recA* internal control.

**Table 2:** **Primers used in this study**.

**Primers**	**Sequence (5′–3′)**
**CONSTRUCTION OF *G. sulfurreducens pilT*- AND *pilB*-DELETION STRAINS**	
pilT1 up MluI 5′	ACACGCGTCTGCGTCATCTTCATCAACCA (MluI)^a^
pilT1 up NdeI 3′	GTCATATGATCGTTCAGTTCCATGGGCTA (NdeI)
pilT1 down NdeI 5′	ACCATATGATCGAGAAGTTCTAGGCCGGT (NdeI)
pilT1 down XbaI 3′	GTTCTAGACAAGAATGGCACTGAGCCCGA (XbaI)
pilT2 up MluI 5′	ACGCGTAATCGCCCTGACCCTCCTGAG (MluI)
pilT2 up NdeI 3′	GTCATATGAAGGTTCATATCCATGGCGGT (NdeI)
pilT2 down NdeI 5′	ACCATATGGCCTTCACCGAGTAGCCGTCC (NdeI)
pilT2 down XbaI 3′	TCTAGAGGGTACCTGAAGGACCACCGT (XbaI)
pilT3 up 5′	GATCTGAGCGCTGTGGTTTC
pilT3 up NdeI 3′	TTATGCGGCCGCCATATGCAGTCGAT (NdeI)
pilT3 down NdeI 5′	GTGTTAACCGGTCATATGCAAAGCCG (NdeI)
pilT3 down 3′	GAGCCGAAGACGTTGGT
pilT4 up 5′	GCTGCGCTTACCGGTCACTTG
pilT4 up NdeI 3′	TTATGCGGCCGCCATATGCAATGCAT (NdeI)
pilT4 down NdeI 5′	GTGTTAACCGGTCATATGCACCTCAG (NdeI)
pilT4 down 3′	CAGAATGACGCCGACGACGATG
pilB up 5′	GATCTGGTCGGATACAACACC
pilB up NdeI 3′	TTATGCGGCCGCCATATGCATCTGCT (NdeI)
pilB down NdeI 5′	GTGTTAACCGGTCATATGCAGTGGCT (NdeI)
pilB down 3′	CTCTTGTGAGGATGCAGGTAC
pCM351 Genta NdeI 5′	TGCATATGGCGGCCGCATAACTT (NdeI)
pCM351 Genta NdeI 3′	TGCATATGACCGGTTAACACGCGTACGTA (NdeI)
**GENETIC COMPLEMENTATION OF *G. sulfurreducens* STRAINS Δ*pilT4* AND Δ*pilB***	
pRG5_pilB_F	CCATGGTTACGAATTCGCCAATACGCCGGAGAGT (EcoRI)
pRG5_pilB_R	TCTTCTTTTCGGATCCCAGATCTCAGATGAGCGGGATT (BamHI)
pRG5_pilT4_F	CCATGGTTACGAATTCTGACATTTTGTAACGGAGAACATC (EcoRI)
pRG5_pilT4_R	TCTTCTTTTCGGATCCTTTCTCCTCCTGTGTACAGATCG (BamHI)
**GENETIC COMPLEMENTATION OF *P. aeruginosa* PAK *pilT*- AND *pilB*-DELETION STRAINS WITH *G. sulfurreducens pilT* AND *pilB* GENES**	
pMMB-RBS-*pilT1*-F	AGGAAACAGAATTCGAGCTCCGAGGAGGATATTCATGGAACTGAACGAT ATCCTCA (SacI)
pMMB-*pilT1*-R	AAACAGCCAAGCTTGCATGCCTAGAACTTCTCGATGCCCTCGA (SphI)
pMMB-RBS-*pilT1*-F	AGGAAACAGAATTCGAGCTCCGAGGAGGATATTCATGGATATGAACCTT CTGTCCCAGA (SacI)
pMMB-*pilT2*-R	AAACAGCCAAGCTTGCATGCCTACTCGGTGAAGGCCAGCT (SphI)
pMMB-RBS-*pilT3*-F	AGGAAACAGAATTCGAGCTCCGAGGAGGATATTCATGGCACGTATCGAC GCACT (SacI)
pMMB-*pilT3*-R	AAACAGCCAAGCTTGCATGCCTACTGGCCCGGCTTTTC (SphI)
pMMB-RBS-*pilT4*-F	AGGAAACAGAATTCGAGCTCCGAGGAGGATATTCATGGCCAACATGCAT CAACT (SacI)
pMMB-*pilT4*-R	AAACAGCCAAGCTTGCATGCTTACCTCATCTGAGGGCGCTGAC (SphI)
pMMB-RBS-*pilB*-F	AGGAAACAGAATTCGAGCTCCGAGGAGGATATTCATGCAGGCTAGCAGA CTGGGAGA (SacI)
pMMB-*pilB*-R	AAACAGCCAAGCTTGCATGCTTAGTCGTCAGCCACGGTAA (SphI)
**qRT-PCR^b^, ^c^**	
RecA660f (primer *1*)	GTGAAGGTGGTCAAGAACAAGGT
RecA737r (primer *2*)	GGAAATGCCCTCACCGTAGTAA
pilT1 RT5′ (primer *3*)	CTTCGAATGCGACACTGC
pilT1 RT3′ (primer *4*)	AATGAAGCGAAACACCATTG
pilT2 RT5′ (primer *5*)	GGTGAGCATCTTCCGTCAG
pilT2 RT3′ (primer *6*)	GGTTGAGTTCCTCGAAGGTC
GSU0231 RT5′ (primer *7*)	CTGCGTCTCACCCTCTTTC
GSU0231 RT3′ (primer *8*)	CATCGTCAGTTTCGCCATAA
GSU0435 RT5′ (primer *9*)	GTGCCGACGTCACCTCTT
GSU0435 RT3′ (primer *10*)	AGCACATCCAGCAGGTAGC
pilT3 RT5′ (primer *11*)	ATGACCCAGTTCAAGAAGGG
pilT3 RT3′ (primer *12*)	GTTGATGAGGTCGATCATGG
pilB RT5′ (primer *13*)	CCATCGACGACATCAAGTTC
pilB RT3′ (primer *14*)	ACTTGTCGATGGCAGTCTTG
pilT4 RT5′ (primer *15*)	ATCGACAAGATCAACACCGA
pilT4 RT3′ (primer *16*)	ACGCAACTCTTGTGAGGATG
pilB RT5′	CCATCGACGACATCAAGTTC
pilB RT3′	ACTTGTCGATGGCAGTCTTG

a *Underlined sequences, restriction enzyme target site; in parentheses, restriction enzyme*.

b *All primers were unique to this study, except for primers RecA660f and RecA737r (Holmes et al., 2005) and primers to make the pilB mutant (Steidl et al., 2016)*.

c *Primer numbers, in parenthesis, are as shown in Figure 1*.

### Heterologous complementation of PAKΔ*pilT* with *G. sulfurreducens pilT* genes

The *P. aeruginosa* strains and plasmids used for heterologous complementation are listed in Table 1. The wild-type PAK strain, the pilin-deficient PAKΔ*pilA* and *pilT*-deficient PAKΔ*pilT* derivatives, and plasmids pMMB67EH and pRK2013 were kindly provided by Matthew C. Wolfgang (University of North Carolina School of Medicine). The *G. sulfurreducens pilT* genes were cloned into the broad host range expression vector pMMB67EH using the In Fusion cloning system (Clontech Laboratories, Inc.). The pMMB67EH plasmids and its derivatives were electroporated in *E. coli* HB101 carrying the mobilizing helper plasmid pRK2013 and transformants were selected on LB agar containing 30 μg/ml carbenicillin and 50 μg/ml kanamycin. The resulting *E. coli* donor strains were mated with the PAKΔ*pilT* strain on antibiotic-free LB agar plates for 2 h at 37°C. After the mating, the cells were suspended in 2 ml of LB medium and serially diluted and plated on LB agar containing 500 μg/ml carbenicillin and 25 μg/ml irgasan. The resulting strains were cultured with 300 μg/ml carbenicillin to maintain the plasmid and, when indicated, with 20 μM IPTG to promote the expression of the cloned genes.

### T4P isolation, biofilm formation, and twitching motility assays using *P. aeruginosa* strains

T4P from all the *P. aeruginosa* strains were purified following a previously described protocol (Castric, 1995). Briefly, 100 μl of an LB culture were plated on triplicate LB-agar plates supplemented with 20 μM IPTG and incubated overnight at 37°C. When needed, carbenecillin (30 μg/ml) was included in the LB-agar medium to maintain the pMMB67EH plasmid or any of its derivatives (Table 1). The cells were scraped off the plate and suspended in 1.5 ml of LB medium and 5 μl of the cell suspension was diluted in 2 ml water to measure its OD_600_ and estimate the cell density. T4P fibers in the cell suspensions were sheared by passing the cells 4 times through a 23-gauge needle and the cells were then removed by centrifugation (2 times at 7500 × g for 15 min at room temperature). The T4P in the supernatant fluids were then precipitated by adding MgCl_2_ to a final concentration of 100 mM and incubating on ice for 60 min. Once precipitated, the T4P were concentrated by centrifugation (12,000 × g for 15 min at 4°C), suspended in 100 μl of 0.1% SDS, and incubated overnight at 4°C to separate the pili bundles. The T4P content in the samples was quantified using the Bio-Rad protein assay Kit (Bio-Rad Laboratories, Inc.), following the manufacturer's recommendations for the standard procedure in microtiter plates, and its concentration (mg/ml) in each sample was normalized by the sample's cell density (in OD_600_ units).

T4P purified in the 0.1% SDS solution were diluted appropriately to standardize for the cell density (OD_600_) and then mixed with an equal volume of Tris-Tricine sample buffer. After incubation at 95°C for 5 min to depolymerize the fibers into the individual pilin subunits, the proteins in 15 μl of sample were separated electrophoretically in a 10–20% Mini-PROTEAN® Tris-Tricine Precast Gel (Bio-Rad). One lane was loaded with 4 μl of the Novex® Sharp Pre-stained Protein Standard (Invitrogen Life Technologies) as standards. The gel was stained overnight with Coomassie Brilliant Blue R-250 Staining Solution (Bio-Rad) and destained overnight with a solution of 50% methanol and 10% acetic acid.

Biofilm assays with strains of *P. aeruginosa* were performed under static conditions in microtiter plates, essentially as described elsewhere (Merritt et al., 2005) but using M63 minimal medium supplemented with 0.4% arginine as the sole carbon and energy source to stimulate biofilm development over a 24 h period (Caiazza and O'Toole, 2004). The medium was supplemented with carbenecillin (30 μg/ml) to maintain the pMMB67EH plasmid and with IPTG (20 μM) to heterologously express the cloned *pilT* genes. The biofilm biomass was then stained with 0.1% crystal violet, and allowed to dry before solubilizing the biofilm-associated dye with 95% ethanol and measuring its OD_580_ (Merritt et al., 2005). To account for technical and biological variability, the biofilm assays included six technical replicates (wells) per strain tested and a minimum of three independent experiments. Outliers were excluded from the compiled biofilm assay results using the Quartile or Fourth-Spread method (Devore, 2000) and the data were plotted as a box-and-whisker graph using the Microsoft Excel software.

For the twitching motility assays, a thin layer (~1 ml) of molten LB medium with 1% agarose and 20 μM IPTG was deposited onto a sterile glass slide and allowed to solidify. The center of the agar was stab-inoculated with overnight LB-20 μM IPTG cultures of the *P. aeruginosa* strains incubated at 37°C with shaking (180 rpm). When needed, carbenecillin (30 μg/ml) was added to the liquid cultures to maintain the pMMB67EH plasmid. After stab-inoculation, the slide was placed inside a Petri dish surrounded by damp, sterile absorbent paper to maintain the humidity inside the chamber and the dish was then sealed with parafilm to prevent drying. After overnight incubation at 37°C, the areas of growth on the surface of the agar were gently removed with a coverslip and the slides were then stained with 1 ml Coomassie Brilliant Blue R-250 Staining Solution (Bio-Rad) for 2 h. The slides were briefly rinsed with 5 ml of a solution of 50% methanol and 10% acetic acid and then destained in 20 ml of the same solution for 4 h before replacing 15 ml of the solution with an equal volume of a solution of 5% methanol and 7.5% acetic acid and incubating overnight. After destaining, the agar layer on the slides was allowed to dry completely before imaging the stained twitching zones with a scanner.

### Construction of *pilT* and *pilB* mutants and complemented strains in *G. sulfurreducens*

*G. sulfurreducens* PCA was used to construct mutants carrying full deletions in each of the four *pilT* homologs and in the *pilB* gene, using the primers listed in Table 2. The general procedure was to replace the full length of the wild-type gene with the *aaaC1* gentamicin cassette of plasmid pCM351 (Marx and Lidstrom, 2002) in the same gene orientation so as to prevent polar effects on downstream genes. For the construction of the *pilT1* and *pilT2* deletions, we PCR-amplified regions approximately 500 bp upstream and downstream of the target genes using the Phusion® high fidelity DNA polymerase (New England Biolabs) and cloned the fragments separately into the pCR2.1-TOPO vector (Invitrogen). The downstream fragments were then digested and cloned into the *Nde*I-*Xba*I site of pCR2.1-TOPO harboring the upstream fragments. This resulted in plasmids carrying the upstream and downstream regions and an *Nde*I site in between. The *aaaC1* gentamicin-resistance cassette was then PCR-amplified from pCM351, digested with *Nde*I, and cloned into the *Nde*I site separating the upstream and downstream fragments. The cloned fragment was excised from the plasmid with *Eco*RI and *Xba*I and gel-purified prior to introduction into electrocompetent cells of *G. sulfurreducens* via electroporation, as described previously (Coppi et al., 2001). The *pilT3, pilT4*, and *pilB* deletion mutants were constructed by recombinant PCR (Coppi et al., 2001), using constructs generated by fusing together the upstream region of the target gene, the *aaaC1* cassette, and the downstream region in a PCR reaction with the external primers. The final PCR product was gel-purified with a Zymoclean Gel DNA Recovery kit (Zymo Research Co.) and incubated at 70°C for 30 min with one volume of 2X GoTaq Green Master Mix (Promega) to produce a 3′ A-overhang prior to cloning into pCR2.1-TOPO. The cloned fragment was PCR-amplified and gel-purified before electroporation into *G. sulfurreducens* electrocompetent cells (Coppi et al., 2001). In all cases, transformants were selected on NBAF plates containing gentamicin (5 μg/ml) and confirmed by PCR, DNA sequencing, and Southern blot hybridization, as described previously (Ausubel et al., 1995).

The pCM158 plasmid was electroporated in the *pilT4* (Δ*pilT4::acc1*) and *pilB* (Δ*pilB::acc1*) mutant strains to express the Cre-recombinase and excise the *acc1* gentamicin-resistance cassette, which is flanked by the *loxP* sites that the Cre protein recognizes and recombines (Marx and Lidstrom, 2002). After confirming the excision of the gentamicin resistance cassette by PCR, the pCM158 plasmid was cured from the strains by culturing in the absence of kanamycin. The resulting strains, designated Δ*pilT4* and Δ*pilB*, were electroporated with plasmids pRG5-*pilT4* and pRG5-*pilB* to express the wild-type *pilT4* and *pilB* genes, respectively, from the medium-copy number plasmid pRG5 (Kim et al., 2005). All cloning steps used the In-Fusion cloning system (Clontech) and selection of transformants was in agar-solidified NBAF medium with spectinomycin (150–300 μg/ml). The Δ*pilT3::acc1* mutation was also introduced in the Δ*pilT4* strain to generate the double *pilT3 pilT4* mutant.

### *G. sulfurreducens* biofilm assays

*G. sulfurreducens* was grown to mid-exponential phase in FWFA medium and serially transferred (10% v/v) three times before diluting the cells in fresh FWAF medium to an OD_600_ of 0.04. Two hundred microliters of this cell suspension were dispensed in the wells of a 96-well plate, which was then sealed to prevent evaporation. The plate was incubated at 30°C inside a TECAN Sunrise™ Absorbance Reader (TECAN, Männedorf, Switzerland) housed in an anaerobic glove bag (Coy Labs) and planktonic growth was monitored periodically (OD_600_) throughout the incubation. After 48 h, the plates were removed from the glove box, the supernatant was decanted, and the biofilms in each well were stained for 20 min with 200 μl of 0.1% crystal violet, gently washed 3 times with 200 μl of ddH_2_O, and air-dried (Stepanovic et al., 2000). The biofilm-associated dye was released from the biofilms by adding 200 μl of 33% (v/v) acetic acid. After 20 min, the plate was mixed on an orbital shaker and the solubilized dye was transferred to a new microtiter plate to record the OD_580_.

### SDS-PAGE and heme staining

Loosely bound proteins of the outer membrane of *G. sulfurreducens* were first mechanically detached from cells grown to mid-exponential phase in FWAF medium. The proteins in the sheared fraction were then separated by SDS-PAGE, as previously described (Cologgi et al., 2011), and those containing heme groups were stained with N, N, N, N-tetramethylbenzidine (Thomas et al., 1976).

### Microbial electrolysis cells (MECs)

The MECs used in these studies were dual-chambered, H-type electrochemical cells equipped with graphite rod (Alfa Aesar,1.27-cm diameter, 99% metals basis, 12 cm^2^) anode and cathode electrodes, separated with a Nafion membrane (Ion Power, Inc., New Castle, DE), and set up as described previously (Speers and Reguera, 2012). Each chamber was filled with 90 ml of anaerobic mineral DB medium (Speers and Reguera, 2012) and an initial concentration of 1 mM sodium acetate was added to the anode chamber as electron donor to support the growth of the anode biofilms and current production. The WT and mutant strains of *G. sulfurreducens* used in the MECs experiments were first grown in DB medium with acetate (20 mM) and fumarate (40 mM) at 30°C, harvested by centrifugation, suspended in DB medium, and inoculated into the anode chamber, as previously described (Speers and Reguera, 2012). A VSP potentiostat (BioLogic, Claix, France) was used to set a 0.24-V potential at the anode electrode vs. a 3 M Ag/AgCl reference electrode (Bioanalytical Systems, Inc.) prior to cell inoculation and to monitor current production at the anode electrode throughout the experiment. The anode electrode was removed from the anode chamber when all the acetate was consumed, as indicated by the drop in current production to < 0.1 mA, and the biofilm cells were then differentially stained (green, live cells; red, dead cells) with the BacLight™ viability kit (Invitrogen), following the manufacturer's recommendations. After staining, the anode electrode was placed on a Lab-Tek® coverglass chamber (Nunc) filled with 3 ml of phosphate buffer saline and imaged with a FluoView FV1000 inverted confocal laser scanning microscope (CLSM) (Olympus) equipped with a UPLFLN 40x oil immersion objective (Olympus, NA 1.30). The green fluorescent dye (SYTO 9) was excited at 488 nm and emission was detected with a BA505-525 band pass filter. The red fluorescent dye (propidium iodide) was excited at 543 nm and emission collected with a BA560IF long pass filter. Vertical image stacks of 200 × 200 μm fields were collected every 1 μm and 3-dimensional image projections were generated with the FV10-ASW 3.0 software (Olympus). The biofilm biomass was estimated from the fluorescence emitted by the biofilm cells using the biovolume function of the COMSTAT software (Heydorn et al., 2000).

## Results

### Description of four *pilT* genes in *G. sulfurreducens*

The genome of *G. sulfurreducens* contains four genes annotated as *pilT* (*pilT1*, GSU0146; *pilT2*, GSU0230; *pilT3*, GSU0436; and *pilT4*, GSU1492) that code for proteins containing the four conserved signature sequences (Walker A and B motifs and Asp and His boxes) of the secretion ATPase protein superfamily that PilT retraction ATPases belong to Savvides (2007). Indeed, all of the PilT paralogs encoded in the genome of *G. sulfurreducens* contain a Walker A motif with a conserved lysine for ATP-binding, a Walker B motif with a conserved glutamate residue for Mg^2+^-binding and ATP hydrolysis, and Asp and His boxes with conserved aspartic and histidine residues, respectively, which are essential for PilT activity (Figure 1; Walker et al., 1982; Savvides, 2007; Chiang et al., 2008; Jakovljevic et al., 2008). As shown in Table 3, the putative PilT proteins are 43–52% identical and 61–70% similar to each other, and PilT3 and PilT4 had the highest identities and similarities to the PilT ATPases that power pilus retraction in *N. gonorrhoeae* (PilT_Ng_), *M. xanthus* (PilT_Mx_), and *P. aeruginosa* strain K (PilT_Pa_) (Whitchurch et al., 1991; Wolfgang et al., 1998; Jakovljevic et al., 2008). PilT3, in particular, was 56% identical and 74% similar to PilT_Pa_, whereas PilT4 was 77% identical and 87% similar to PilT_Mx_.

**Figure 1:**
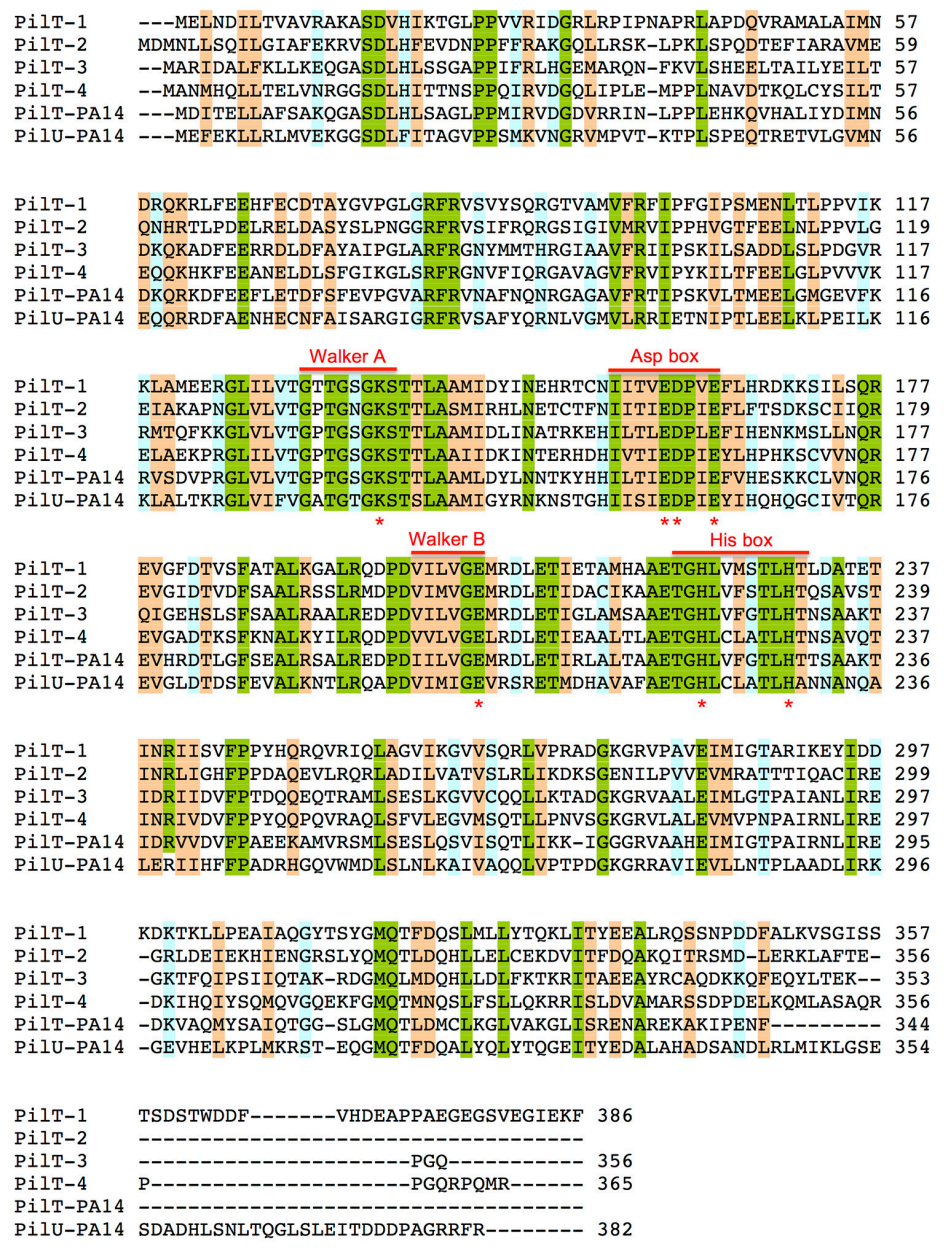
**Amino acid sequence alignments of PilT proteins from *G. sulfurreducens* and *P. aeruginosa* PA14**. Multiple sequence alignment analysis was carried out using Clustal O (1.2.2) program at the UniProt database (http://www.uniprot.org). Shaded areas show positions in the alignment with a single, fully conserved residue (green) or with conservation between groups of strongly (orange) or weakly (blue) similar properties (Gonnet PAM 250 matrix scoring >0.5 or ≤ 0.5, respectively). The conserved Walker A and Walker B motifs and Asp and His boxes of secretion ATPases are indicated. Red stars under the sequence alignment mark highly conserved lysine (Walker A), glutamate (Walker B), aspartate and/or glutamate (Asp box) and histidine (His box) residues that are essential for PilT activity.

**Table 3:** **Identity and similarity of the putative PilT proteins of *G. sulfurreducens* (in red) in pairwise comparisons with each other and the PilT's from *N. gonorrhoeae* (PilT_Ng_), *M. xanthus* (PilT_Mx_), and *P. aeruginosa* strain K (PilT_Pa_)**.

**Identity (%)/*Similarity*^a^ (%)**	**PilT1**	**PilT2**	**PilT3**	**PilT4**	**PilT_Ng_**	**PilT_Mx_**	**PilT_Pa_**
PilT1		*64*	*68*	*70*	*67*	*70^b^*	*67*
PilT2	47		*61*	*64*	*62*	*62*	*61*
PilT3	50	43		*67*	*73*	*67*	*74*
PilT4	52	44	49		*68*	*87*	*68*
PilT_Ng_	45	42	55	49		*67*	*82*
PilT_Mx_	51	43	49	77	45		*71*
PilT_Pa_	48	45	56	52	67	54	

a *Similarity values (above black boxes) are italicized*.

b *Highest identities or similarities for each of the G. sulfurreducens PilT homologs compared to themselves and to the other bacterial PilT proteins are highlighted in gray*.

**Figure 2:**
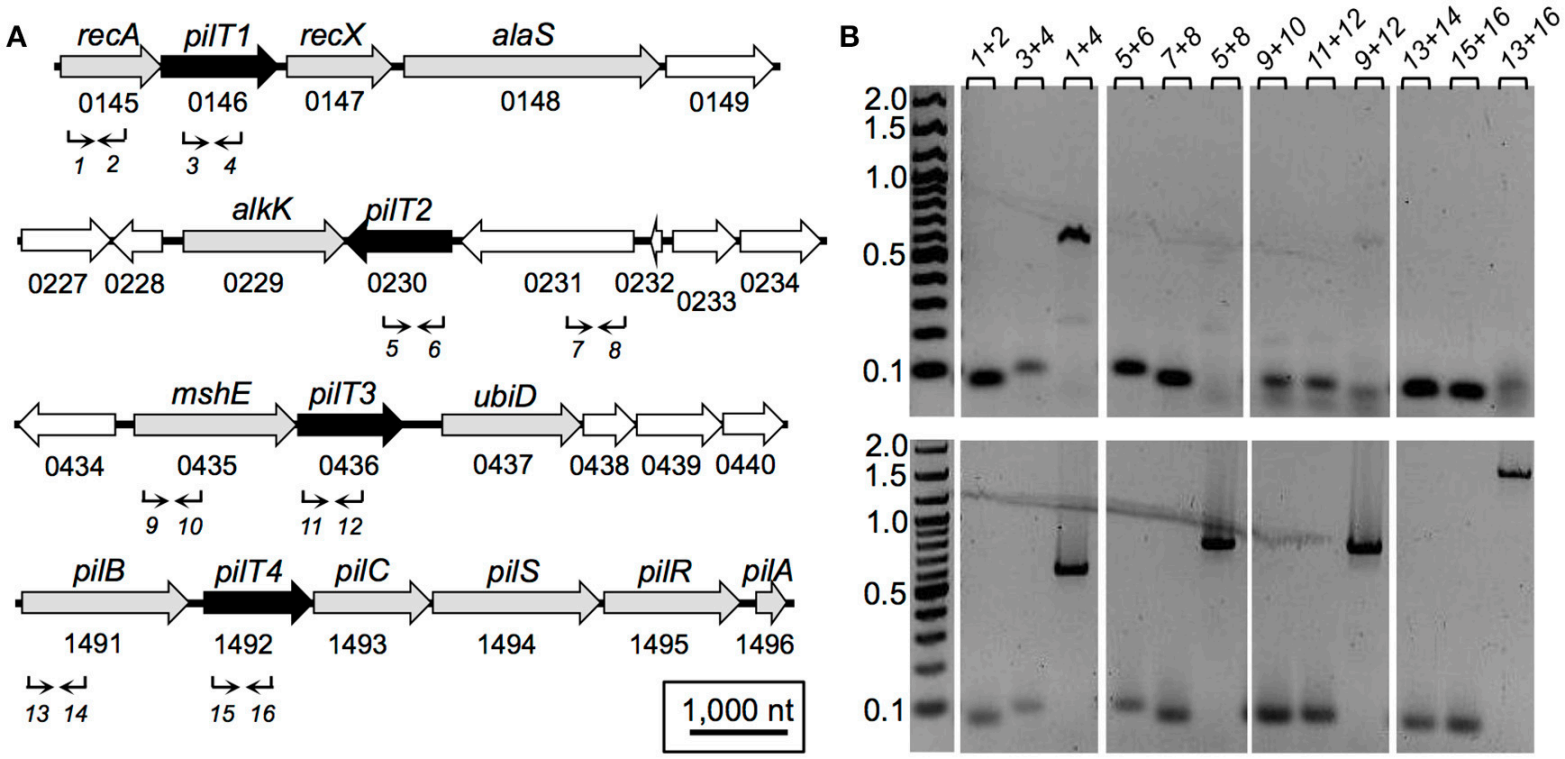
**Genetic (A) and transcriptional (B) organization of *pilT* genes in *G. sulfurreducens***. **(A)** Schematic view of the genomic region containing each of the four *pilT* genes (labeled 1–4, black arrows) and neighboring ORFs (white arrows, hypothetical gene; gray arrows, annotated function). Also shown are gene designations (gene name and ORF number, above and below, respectively) and PCR primers (*1*–*16*; Table 2) and direction of amplification (arrows). **(B)** Agarose gels showing cDNA amplified from each *pilT* gene region and neighboring genes (top panels) in reference to products amplified from genomic DNA controls (bottom panels). PCR-amplifications used primer sets *1*–*16*, as indicated in **(A)** and listed on top of the gel panels in **(B)**. Lanes, gene target (primer combination): *recA* (*1*+*2*), *pilT1* (*3*+*4*), *recA-pilT1* (*1*+*4*), *pilT2* (*5*+*6*), *GSU0231* (*7*+*8*), *pilT2-GSU0231* (*5*+*8*), *mshE* (*9*+*10*), *pilT3* (*11*+*12*), *mshE-pilT3* (*9*+*12*), *pilB* (*13*+*14*), *pilT4* (*15*+*16*), *pilB-pilT4* (*13*+*16*).

The genetic arrangement of the *pilT* genes in *G. sulfurreducens* and its transcriptional expression profile with neighboring genes is shown in Figure 2. *pilT1* (GSU0146) is flanked by the *recA* and *recX* genes (Figure 2A), a gene arrangement that is conserved in other sequenced genomes in the *Geobacteraceae* family (*Geobacter metallireducens, Geobacter uraniireducens*, and *Pelobacter carbinolicus*). Furthermore, *pilT1* was co-transcribed with the *recA* gene (Figure 2B), consistent with a role for PilT1 in the cellular recombination machinery. The *pilT2* gene (GSU0230), on the other hand, is located downstream of a gene (GSU0231) encoding a hypothetical protein (Figure 2A) but transcribed independently from it under the conditions tested (Figure 2B). Downstream of *pilT2*, and in opposite orientation, is the *alkK* gene (GSU0229), which encodes a putative medium-chain fatty acid CoA ligase. This arrangement is unique to *G. sulfurreducens*. Further, *pilT2* was not conserved in all of the *Geobacter* spp. with sequenced genomes. We found, for example, a *pilT2* homolog in *G. metallireducens* (Gmet0260) but none in *G. uraniireducens*. The lack of conservation of *pilT2* in other *Geobacter* genomes contrasts with the high conservation of the pilin-encoding gene, *pilA* (Reguera et al., 2005) and argues against its role as the PilT ATPase of the T4P apparatus in these bacteria.

The *pilT3* gene (GSU0436) is downstream of a gene (*mshE*) encoding a putative mannose-sensitive haemagglutinin (MSHA) biogenesis/general secretory system II protein that is homologous to the pilin polymerization ATPase of MSHA pili in *Vibrio cholerae* (Jones et al., 2015). The *mshE pilT3* arrangement is conserved in the genomes of *G. metallireducens* and *G. uraniireducens*. The two genes are unlikely to be co-transcribed in *G. sulfurreducens* because, although reverse transcription produced a faint band spanning both genes, the band was smaller than in the controls amplified from genomic DNA and thus it was more likely the product of non-specific binding of one or both primers (Figure 2B).

**Figure 3:**
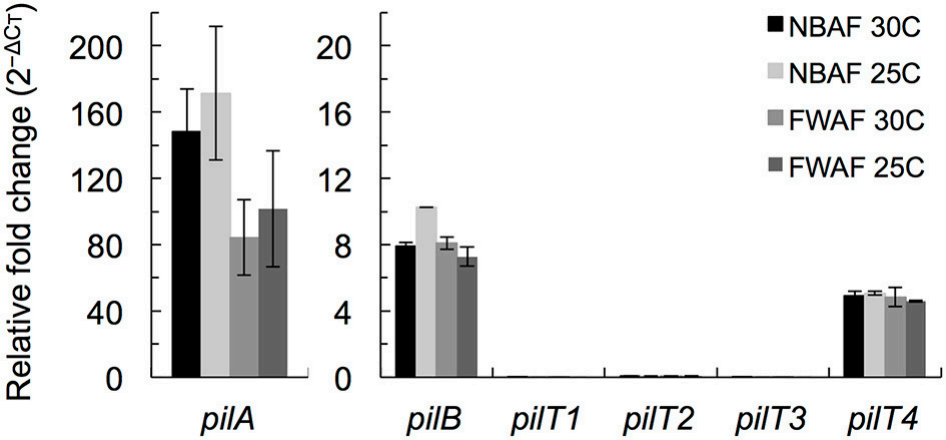
**Transcriptional expression analysis of *pil* genes in *G. sulfurreducens* by qRT-PCR**. The expression levels of *pilA, pilB*, and the four *pilT* (*pilT1-4*) genes of *G. sulfurreducens* were measured in duplicate samples in reference to *recA* controls in cultures grown in rich NBAF and mineral FWAF media incubated at pili-inducing (25°C) or non-inducing (30°C) temperatures. There were no statistically significant changes (*p* < 0.01) in the relative expression of the target genes in reference to the pili-inducing conditions (FWAF 25C) in two-tailed pairwise comparisons using the *t*-test function of the Microsoft® Excel® software.

The *pilT4* gene (GSU1492) is flanked by genes encoding proteins with predicted or confirmed roles in pili biogenesis and its regulation such as the pilin assembly ATPase PilB (Steidl et al., 2016), the pilin biogenesis protein PilC, the PilS sensor, the PilR transcriptional regulator (Juarez et al., 2009), and the pilin subunit PilA (Reguera et al., 2005; Figure 2A). This genetic arrangement is conserved in other *Geobacteraceae* (*G. metallireducens, G. uraniireducens*, and *P. carbinolicus*) and is similar to the organization of the *pil* region of *M. xanthus* (Wall and Kaiser, 1999). The genes flanking *pilT4* were also very conserved among *Geobacter* species. The PilB protein encoded by GSU1491 was, for example, 94% identical and 97% similar to Gmet1393 from *G. metallireducens*, and 88% identical and 96% similar to Gura2682 from *G. uraniireducens*. Similarly, the PilC protein encoded by GSU1493 was 89% identical and 95% similar to Gmet1395 from *G. metallireducens*, and 81% identical and 90% similar to Gura2680 from *G. uraniireducens*. PilB and PilC proteins are the most highly conserved components of the bacterial T4P apparatus and related systems such as the bacterial type II secretion system and archaeal motility systems and both have been proposed to form the minimal functional motor unit of the pilus (Burrows, 2012). Reverse Transcription (RT)-PCR analysis using a primer set spanning the intergenic *pilB pilT4* failed to amplify a full-length product, suggesting that, under the conditions tested, the two genes are not co-transcribed (Figure 2B). Yet, despite their independent transcription, the expression profiles of *pilB* and *pilT4* in both rich (NBAF) and mineral (FWAF) medium were similar and matched well the expression profile of *pilA* regardless of the incubation temperature (30°C or pili-expressing temperatures of 25°C; (Reguera et al., 2005; Cologgi et al., 2011; Figure 3). Indeed, the *pilT4, pilB*, and *pilA* genes were up-regulated under all of the conditions tested, whereas *pilT1, pilT2*, and *pilT3* were all down-regulated. Hence, based on gene and protein homologies, gene clustering, overall conservation in other *Geobacter* spp., and expression profiles with other *pil* genes, *pilT4* is arguably the most likely candidate to encode for the PilT motor of the T4P apparatus in *G. sulfurreducens*.

### PilT3 and PilT4 rescue pili retraction defects in *P. aeruginosa*

**Figure 4:**
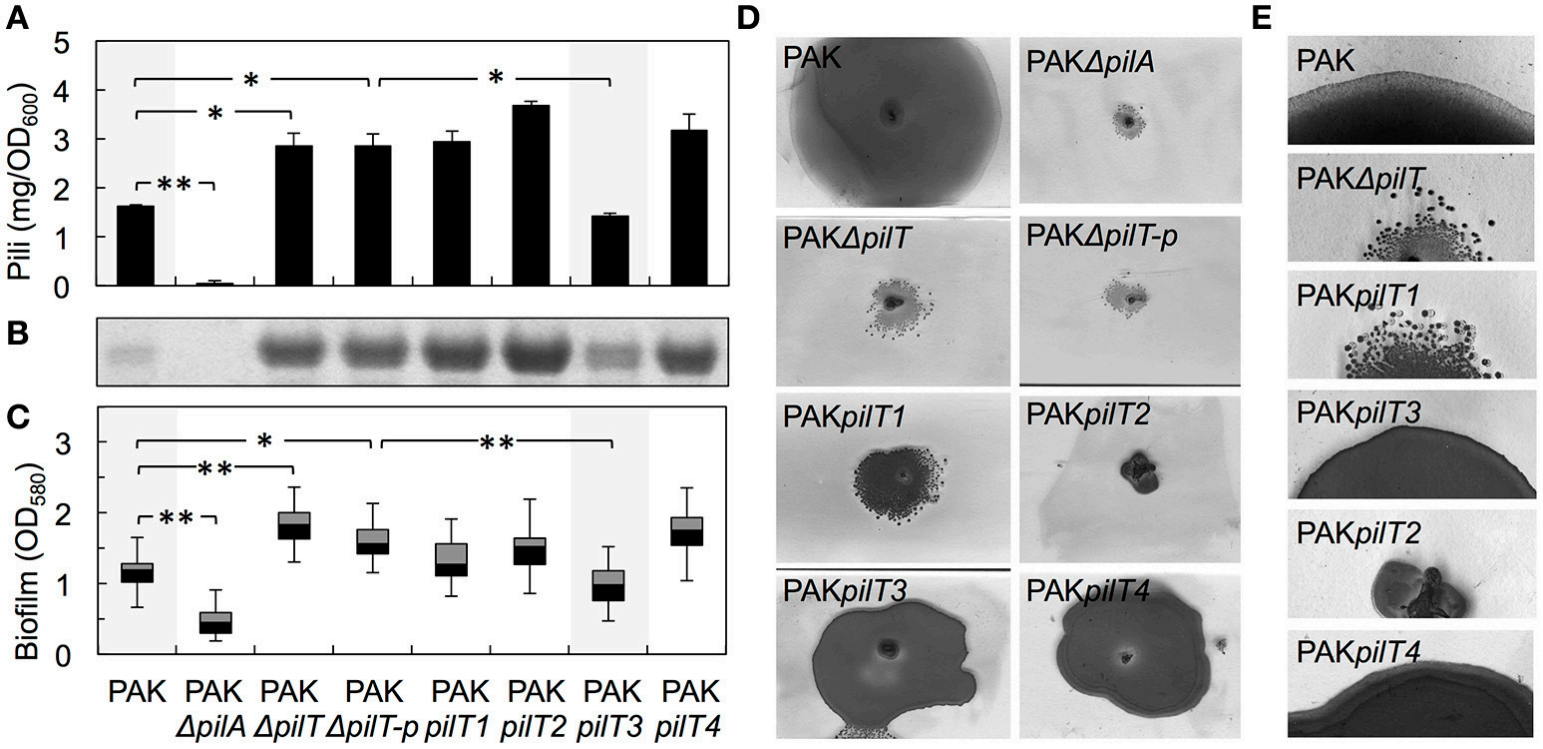
**Heterologous expression of *G. sulfurreducens pilT* genes in the hyperpiliated, non-twitching PAKΔ*pilT* mutant. (A–C)** Restoration by *pilT3* of WT levels of piliation [shown as purified pili protein **(A)** and Coomassie-stained pilin protein band after SDS-PAGE **(B)**] and biofilm density **(C)** in the PAKΔ*pilT* strain. The pilin-deficient PAKΔ*pilA* and hyperpiliated PAKΔ*pilT*-p strain (carrying the empty pMMB67EH vector) are shown as controls. Data are average and standard deviation of pili protein (mg/OD_600_) purified from three biological replicates **(A)** and median and first and third quartiles of biofilm biomass from six biological replicates, each containing 5–8 technical replicates **(C)**. Statistically significant changes in pili content **(A)** and biofilm biomass **(C)** were determined in two-tailed pairwise comparisons with the WT or PAKΔ*pilT*-p strains using the *t*-test function of the Microsoft Excel software (^*^p < 0.01, ^**^p < 0.001). **(D)** Twitching motility slide assays showing the Coomassie-stained zones of surface expansion in the wild-type PAK, which are absent in the pilin-deficient PAKΔ*pilA* and non-twitching PAKΔ*pilT* and PAKΔ*pilT*-p strains. Expression of *pilT3* and *pilT4* (PAK*pilT3* and PAK*pilT4* strains) restored the twitching motility deficiency of the PAKΔ*pilT* strain. **(E)** Micrographs showing the zones of expansion on the edge of PAK colonies shown in **(D)**.

We gained insights into the functionality of each of the PilT proteins encoded in the genome of *G. sulfurreducens* in heterologous complementation experiments that tested the ability of each *pilT* gene to complement the phenotypes associated with a mutant of *P. aeruginosa* strain K (PAK) carrying an in-frame deletion in the *pilT* gene (PAKΔ*pilT*). The inability of the PAKΔ*pilT* mutant to retract the T4P leads to hyperpiliation, a phenotype that can be restored by genetic complementation of the mutation with the wild-type *pilT* copy *in trans* (Sundin et al., 2002). PilT3 was the only PilT homolog of *G. sulfurreducens* that rescued the hyperpiliated phenotype of the PAKΔ*pilT* mutant (*p* < 0.01), reducing the piliation per cell to WT levels (Figures 4A,B). The hyperpiliation of the PAKΔ*pilT* mutant also increases the adhesion of the cells to surfaces and co-aggregation and promotes the formation of denser biofilms under static conditions, a phenotype that is restored by genetic complementation (Chiang and Burrows, 2003). We observed a similar restoration of the biofilm phenotype when the PAKΔ*pilT* strain was genetically complemented with the *pilT3* gene of *G. sulfurreducens* (Figure 4C). Thus, the PilT3 protein of *G. sulfurreducens* is able to power pilus retraction and control the levels of piliation and biofilm formation in *P. aeruginosa*.

Twitching motility in *P. aeruginosa* requires the activity of PilT and its paralog, PilU (Whitchurch et al., 1991; Whitchurch and Mattick, 1994). This mode of surface translocation involves cycles of pilus extension, adhesion of the pilus tip to the surface, and retraction of the pilus fiber to pull the cell (Skerker and Berg, 2001). The PilT motor hydrolyzes ATP to energize the retraction of the surface-attached pilus and provides the force needed to move the cell forward (Merz et al., 2000). As a result, the inactivation of PilT results in a non-twitching phenotype (Whitchurch and Mattick, 1994). Thus, we also investigated the ability of the PilT proteins of *G. sulfurreducens* to restore the twitching motility defect of the PAKΔ*pilT* mutant in subsurface twitching motility assays on glass slides (Figure 4D). Interestingly, not only PilT3 but also PilT4 rescued the non-twitching phenotype (Figure 4D). Examination of the colony edges at higher magnification (Figure 4E) revealed the smooth and thin expansion zones in the strains genetically complemented with *pilT4*, and to a lesser extent with *pilT3*, that are characteristic of the WT twitching motility phenotype (Semmler et al., 1999). Thus, the heterologous expression studies demonstrated that both PilT3 and PilT4 can function as motor ATPases in *P. aeruginosa*, but PilT4's activity is restricted to twitching motility.

### Inactivation of PilT4, but not PilT3, in *G. sulfurreducens* promotes the formation of denser biofilms

**Figure 5:**
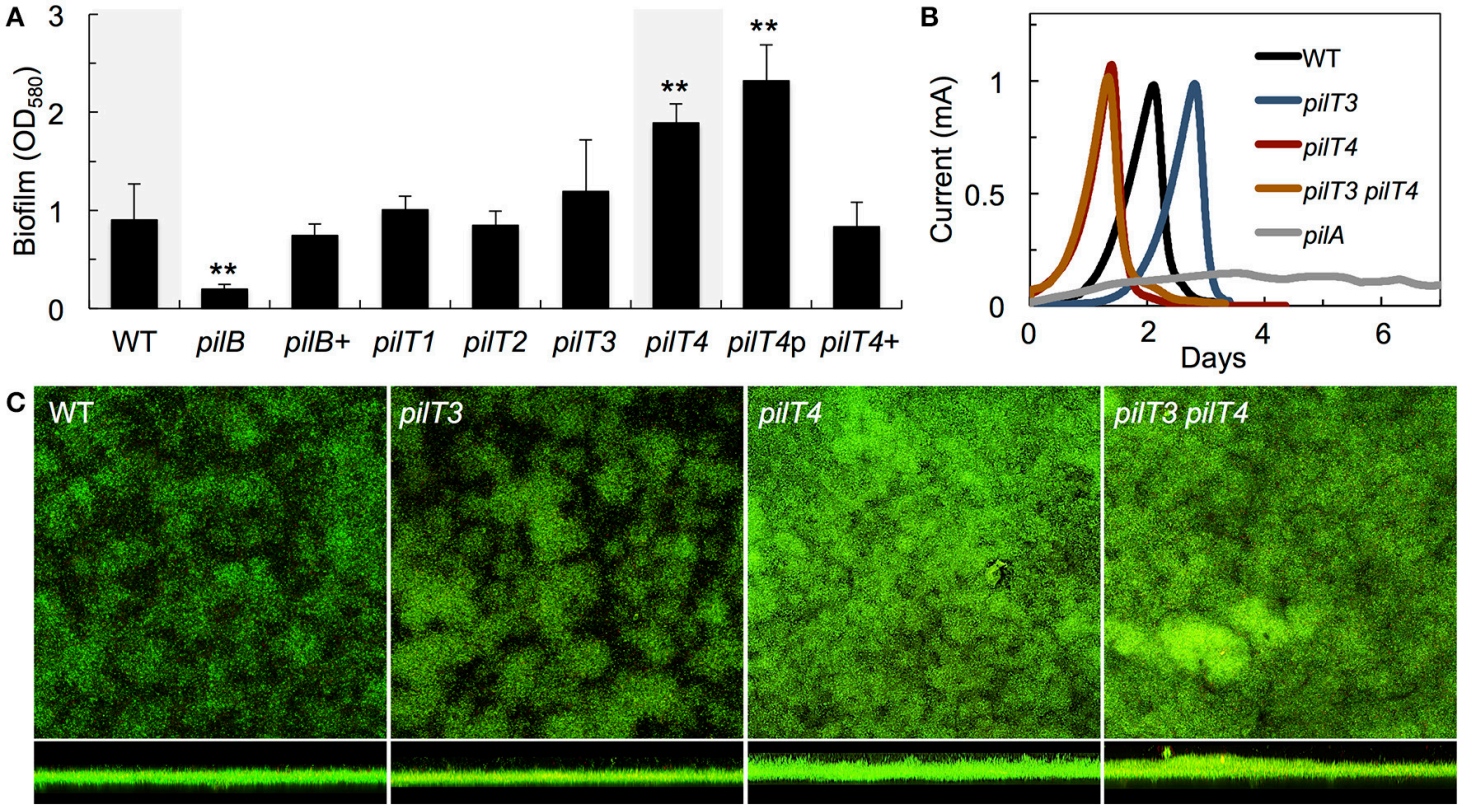
**Biofilm formation on plastic surfaces (A) and on poised electrodes (B,C) by WT and *pilT* mutants of *G. sulfurreducens***. **(A)** Biomass of 48-h biofilms of a pilus-deficient *pilB* mutant, its complemented strain *pilB*+, and mutants carrying a deletion in one of the *pilT* paralogs (*pilT1*-*4*) relative to the WT strain. The biofilm biomass of a genetically complemented *pilT4*+ strain and *pilT4*p control strain carrying the empty plasmid are also shown. Data are the average and standard deviation of four biological replicates, each containing six technical replicates. Statistically significant changes were determined in two-tailed pairwise comparisons with the WT or PAKΔ*pilT*-p strains using the *t*-test function of the Microsoft® Excel® software (^**^p < 0.001). **(B)** Current production by anode biofilms in MECs fed an initial concentration of 1 mM acetate and driven by the WT, *pilT3, pilT4*, and *pilT3 pilT4* strains in reference to the pilin-deficient mutant *pilA*. **(C)** Confocal micrographs (200 × 200 μm2) showing top and side view projections of current-producing anode biofilms collected from MECs in **(B)** and stained with the BacLight viability dies (green, live cells; red, dead cells).

As in *P. aeruginosa*, the pili of *G. sulfurreducens* also promote cell-cell aggregation and biofilm formation (Reguera et al., 2007; Cologgi et al., 2014). Thus, we investigated the biofilm-forming abilities of *G. sulfurreducens* mutants carrying *pilT* deletions in reference to the WT strain (Figure 5A). As controls we also included a pili-defective *pilB* mutant, which makes thin biofilms, and its genetically complemented strain *pilB*+, which expresses the wild-type *pilB* gene *in trans* and restores pili assembly and biofilm formation (Steidl et al., 2016). Deletion of *pilT1, pilT2*, and *pilT3* had no significant effect on biofilm formation in *G. sulfurreducens*. By contrast, a *pilT4* mutant formed denser biofilms on plastic surfaces (*p* < 0.001), a phenotype associated with hyperpiliation in this bacterium (Reguera et al., 2007; Cologgi et al., 2014). The enhanced biofilm phenotype of the *pilT4* mutant was restored in the genetically complemented strain, *pilT4*+, but not in the control *pilT4*p strain, which carries the empty pRG5 expression vector in the same *pilT4* mutant background (Figure 5A). The enhanced biofilm-forming abilities of the *pilT4* mutant cannot be explained by differences in cell growth, because planktonic growth rates for this mutant were comparable to those measured in all of the strains tested (~0.12 OD_660_/h). Rather, the dense biofilm phenotype observed in the *pilT4* mutant is consistent with the increased adhesion and co-aggregation reported for hyperpiliated strains of *G. sulfurreducens* (Reguera et al., 2007) and other bacteria (Deziel et al., 2001; Chiang and Burrows, 2003).

### PilT4 influences the structure of anode biofilms of *G. sulfurreducens* and reduces the levels of current production per cell

The T4P of *G. sulfurreducens* are also required for biofilm formation on anode graphite electrodes poised at a metabolically oxidizing potential and for optimal current production in MECs (Reguera et al., 2006; Steidl et al., 2016). Hence, we grew the *pilT3* and *pilT4* mutants in the anode chamber of a MEC fed with 1 mM acetate and monitored current production in reference to the WT strain (Figure 5B). The *pilT4* mutant attached rapidly to the electrode and initiated current production before any other strain. The average attachment or *lag* phase in triplicate MECs for the *pilT4* mutant was, for example, 5 ± 1 h, which is 4–5 times shorter than the WT (24 ±11 h). By contrast, the *pilT3* mutant was delayed in attachment (33 ± 3 h average *lag* phase in triplicate MECs; Figure 5B). Yet once the cells attached and the exponential phase of biofilm growth started (corresponding to the linear phase of current production) the *pilT4* and *pilT3* mutant biofilms produced current at rates comparable to the WT strain and also reached similar levels of current maxima (Figure 5B). This contrasts with the severe defect in current production of the *pilA* mutant (Figure 5B), which carries a deletion in the pilin-encoding gene *pilA* that prevents it from making conductive pili (Reguera et al., 2005; Cologgi et al., 2011) and is also unable to secrete the matrix-associated cytochrome OmcZ_*S*_ required for electron transfer to the underlying electrode (Steidl et al., 2016). Furthermore, coulombic efficiencies in the WT and *pilT4* biofilms were similar (80–90%), indicating that on average the WT and *pilT4* anode biofilms converted the same amount of acetate into electricity (80–90%). However, the *pilT4* mutant accumulated a much greater biofilm biomass on the electrode (23.3 ± 4.2 μm^3^/μm^2^) than the WT (11.9 ± 1.5 μm^3^/μm^2^) to produce the same amount of current as the WT (Figure 5C). This indicates that, on average, less current was generated per cell in the *pilT4* biofilms than in the WT biofilms.

We also ruled out compensatory effects between PilT3 and PilT4 by testing the ability of a *pilT3 pilT4* double mutant to colonize and generate current in the MECs. The double mutant was phenotypically indistinguishable from the *pilT4* single mutant, having shorter *lag* phases but otherwise producing current like the WT (Figure 5B) and forming denser biofilms on the anode electrodes (Figure 5C). Hence, the *pilT4* mutation was dominant over *pilT3*, not only rescuing the colonization defect of the *pilT3* cells, but also promoting faster attachment and the formation of denser biofilms. This is again consistent with the aggregative nature of hyperpiliated strains of *G. sulfurreducens* (Reguera et al., 2007; Cologgi et al., 2014), which is expected for cells defective in pilus retraction (Sundin et al., 2002).

### PilT4 is required for Fe(III) oxide reduction in *G. sulfurreducens*

**Figure 6:**
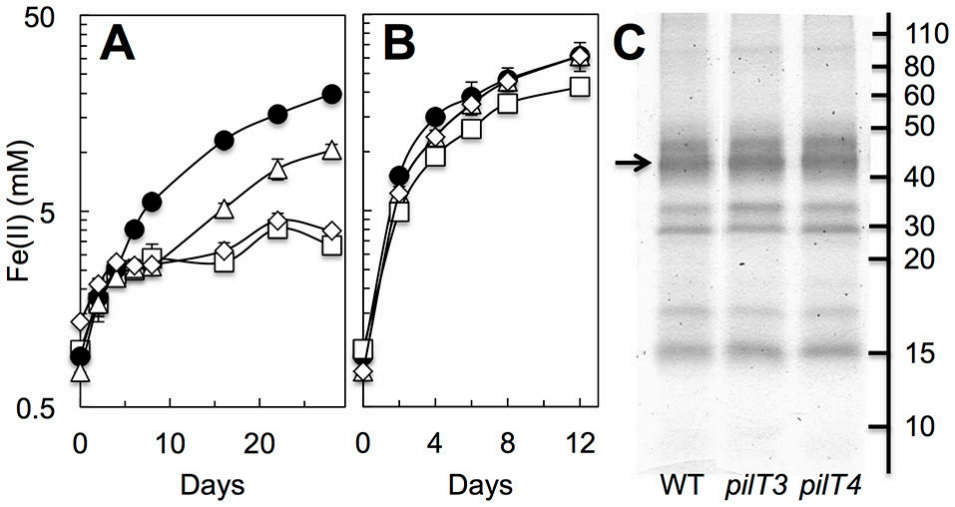
**Role of PilT3 and PilT4 in extracellular electron transfer to Fe(III) oxides. (A,B)** Fe(III) oxide reduction (measured as acid-extractable Fe[II]) in the absence **(A)** or presence **(B)** of the metal chelator NTA (4 mM) by the *pilT3* (triangles), *pilT4* (squares), and double *pilT3 pilT4* (rhomboid) mutants of *G. sulfurreducens* in reference to the WT (solid circles). Shown are the average and standard deviation of triplicate cultures for each strain. **(C)** Heme-stained proteins mechanically detached from the outer membrane of the WT, *pilT3*, and *pilT4* strains. The arrow points at the migration of the OmcS cytochrome.

As *G. sulfurreducens* also expresses pili to access and reduce Fe(III) oxides (Reguera et al., 2005), we tested the ability of the *pilT3, pilT4*, and double *pilT3 pilT4* mutants to grow with Fe(III) oxides when provided as sole electron acceptor in reference to the WT strain (Figure 6A). The strains were first grown in Fe(III) oxide media with 4 mM of NTA, a metal chelator that solubilizes the Fe(III) from the Fe(III) oxides and alleviates the need for cells to directly contact the oxide minerals (Reguera et al., 2005). Exponentially growing cells were then transferred to Fe(III) oxides medium without NTA. The chelating activities of the NTA carried over in the inoculum (ca. 0.4 mM) stimulated initial growth in the cultures, as indicated by the low levels of reduced iron (Fe[II]) solubilized by all of the strains during the first 4–5 days (Figure 6A). However, whereas the WT continued to grow exponentially beyond this initial phase with similar doubling times (2.9 ± 0.1 days), the *pilT3* mutant plateaued for approximately 4 days before resuming Fe(III) reduction at rates similar to the WT strain (Figure 6A). This contrasts with a Tyr3 mutant, which carries alanine replacements in the pilin's three tyrosines that increase the electrical resistance of the T4P (Steidl et al., 2016) but is able to resume exponential growth without delay after the NTA-dependent phase, albeit at slower rates (doubling time, 10.5 ± 0.8 days). The delay observed in the *pilT3* mutant is consistent with a strain that has a transient defect in adhesion, as we previously observed during the colonization of anode electrodes in MECs (Figure 5B). However, the *pilT3* defect was chemically rescued in the presence of the metal chelator NTA, which provided a soluble, reducible form of Fe(III) for cellular respiration throughout the experiment (Figure 6B). By contrast, the reductive activities of the *pilT4* mutant never fully recovered after the NTA-dependent phase (Figure 6A). Furthermore, as we observed during the colonization of electrodes (Figure 5B), introducing the *pilT3* mutation in the *pilT4* mutant (*pilT3 pilT4* mutant) did not change the *pilT4* mutant phenotype. Thus, they are unlikely to have complementary roles or compensate for each other's inactivation. And, as with *pilT3*, the defects of the single *pilT4* and double *pilT3 pilT4* mutants were chemically rescued with NTA (Figure 6B).

We also ruled out pleiotropic effects of the mutations in *c*-cytochrome expression by examining the type and abundance of outer membrane *c*-cytochromes (Figure 6C). There were no measurable differences in the heme-containing proteins mechanically sheared from the outer membrane of the WT, *pilT3*, and *pilT4* strains and all included the OmcS *c*-cytochrome that is required for optimal reduction of Fe(III) oxides (Mehta et al., 2005). Hence, the Fe(III) oxides experiments support our early conclusion that PilT4 is the retraction ATPase in *G. sulfurreducens* and highlight a previously unrecognized role for pilus retraction in the respiration of Fe(III) oxides.

## Discussion

The clustering of *pilT4* with other *pil* genes (Figure 2), including the pilin-encoding gene *pilA* and the gene encoding the pilin assembly ATPase PilB, as well as the similar expression profiles of this *pilT* paralog and other *pil* genes (Figure 3) and conservation in *Geobacter* spp., makes PilT4 the most obvious candidate to power T4P retraction in *G. sulfurreducens*. We demonstrated its functionality through its ability to restore the twitching motility phenotype of PAKΔ*pilT*, the retraction-deficient mutant of *P. aeruginosa* strain K (Figures 4D,E). Thus, although PilT4 was less similar (68%) to PilT_*Pa*_ than PilT3 (74%) (Table 3), it was also recruited to the T4P apparatus of *P. aeruginosa* and it was able to hydrolyze ATP to generate the force needed to power pilus retraction during twitching motility (Merz et al., 2000; Skerker and Berg, 2001; Maier et al., 2002). Yet PilT4 did not restore WT levels of piliation in planktonic cultures (Figures 4A,B) nor did it genetically complement the biofilm phenotype of the PAKΔ*pilT* mutant in static biofilm assays (Figure 4C). Although the exact composition of the T4P apparatus of *P. aeruginosa* is not known, PilB, PilC, and PilT have been proposed to form the minimal functional motor unit (Burrows, 2012). PilC is predicted to interact with both PilB and PilT in the motor complex to coordinate antagonistic cycles of polymerization and depolymerization, respectively (Takhar et al., 2013). This minimal motor requires an additional component, PilU, for twitching motility (Whitchurch and Mattick, 1994) but not for biofilm formation under static conditions (Chiang and Burrows, 2003). Studies suggest that differences in just a few key residues of the PilT protein can induce conformational changes such that prevent productive interactions with other components of the T4P apparatus (Nakasugi et al., 2007). Hence, the PilT4 conformation may promote interactions with the T4P apparatus of *P. aeruginosa* formed for twitching motility, which includes PilU_*Pa*_, but may not be efficiently recruited by the T4P motor unit that powers pilus retraction in planktonic cells and during biofilm formation, which does not include PilU_Pa_.

Consistent with its role as a PilT motor, inactivating the *pilT4* gene in *G. sulfurreducens* led to the formation of the dense biofilms (Figure 5) that are characteristic of the enhanced aggregated behavior of hyperpiliated strains in this bacterium (Reguera et al., 2007; Cologgi et al., 2014). Furthermore, *pilT4* cells colonized the surface of anode electrodes faster than the WT strain (Figure 5B). The initial phase of colonization comprises the period whereby planktonic cells attach to the anode to form a saturating monolayer (Marsili et al., 2010). The attached cells then express key biofilm components such as the conductive pili (Reguera et al., 2006; Steidl et al., 2016), the biofilm exopolysaccharide (EPS) (Rollefson et al., 2011), and the matrix-associated *c*-cytochrome OmcZ_S_ (Nevin et al., 2009) and grow and divide exponentially while coupling the oxidation of the electron donor (e.g., acetate) to the reduction of the anode electrode (Marsili et al., 2010). Current production increases linearly during this phase of exponential growth until acetate availability becomes growth-limiting, which marks the beginning of the phase of current deceleration (Speers and Reguera, 2012). Hence, the shorter *lag* phase observed in the *pilT4* mutant is consistent with a mutant with enhanced adhesive properties, as reported for hyperpiliated PilT-deficient strains (Chiang and Burrows, 2003). Hyperpiliated strains of *G. sulfurreducens* also have enhanced co-aggregation and, as a result, form denser biofilms (Reguera et al., 2007; Cologgi et al., 2014), a phenotype that also matches well the dense anode biofilms observed in the *pilT4*-driven MECs (Figure 5C).

Interestingly, although more *pilT4* cells accumulated on the anode electrodes than in the WT biofilms (Figure 5C), coulombic efficiencies and parameters used to measure the biofilm electroactivity such as rates of current production and maximum current were similar in the *pilT4* and WT MECs (Figure 5B). Thus, although the *pilT4* biofilms converted the same amount of acetate into current over the same period of time, less current was generated on a per cell basis than in the WT biofilms. The reduced efficiency of individual *pilT4* cells in the anode biofilms cannot be explained by defects in *c*-cytochrome expression, as reported for other strains with pili defects (Juarez et al., 2009; Cologgi et al., 2011), because the profile and expression levels of outer membrane heme-containing proteins was similar in the WT and *pilT4* cells (Figure 6C). The conductive pili network works coordinately with *c*-type cytochromes of the biofilm matrix to maintain optimal rates of electron transfer in anode biofilms (Steidl et al., 2016). The inability of the *pilT4* biofilm cells to maintain optimal pilus dynamics through antagonistic cycles of pilus extension and retraction may reduce the number of productive interactions between the pili and electron carriers of the biofilm matrix, thus reducing the rates of electron transfer per cell and requiring the formation of dense biofilms to compensate for the defect. Oral bacteria, which like *G. sulfurreducens* also use pili to co-aggregate and adhere to surfaces, rely on dynamic cycles of pilus extension and retraction to maintain optimal cell-cell distances needed for effective cell-cell communication and coordinated behavior (Kolenbrander et al., 2010). Cell-cell distance is particularly important for electron transfer in electroactive biofilms, which rely on multistep electron hopping among the electron carriers of the biofilm matrix (Strycharz-Glaven et al., 2011; Yates et al., 2015; Steidl et al., 2016). Hence, PilT4-mediated pilus retraction may be important to maintain optimal cell-cell separation and to promote interactions between the pili and other electron carriers in the biofilm matrix.

Our studies also provide evidence for a role of PilT4-mediated pilus retraction during the respiration of Fe(III) oxides. Inactivation of PilT4 prevented the cells from reducing the oxide minerals unless in the presence of NTA (Figure 6), a metal chelator that alleviates the need for iron-reducers to directly contact the mineral (Lovley et al., 1994; Lovley and Woodward, 1996; Manzella et al., 2013). As a result, NTA chemically rescues defects in pili synthesis (Reguera et al., 2005) and conductivity (Feliciano et al., 2015) in *G. sulfurreducens*. The inability of the *pilT4* mutant to reduce Fe(III) oxides via a direct-contact mechanism contrasts with earlier reports (Reguera et al., 2005) indicating that PilT4 inactivation does not affect Fe(III) oxides reduction. These early studies required extended incubation times (60 days or longer) to unmask mutant phenotypes during the reduction of Fe(III) oxides, which could have selected for strains carrying compensatory mutations (Leang et al., 2005). We minimized the selection of compensatory phenotypes by first growing all of the strains in cultures with Fe(III) oxides in the presence of the metal chelator NTA. Furthermore, some NTA was carried over with the inoculum, which stimulated the initial growth of all the strains until the concentrations of NTA per cell became growth-limiting. Beyond this point, the cells depend on the proper functioning of the pili to bind and reduce the oxides and pili-defective phenotypes are unmasked. The *pilT4* mutation caused the most severe defect, as indicated by the inability of the mutant to reduce the Fe(III) oxides beyond the NTA-mediated phase (Figure 6A). However, the mutant phenotype was chemically rescued when an excess NTA was provided as a mediator (Figure 6B) to bypass the cells requirement to use pili to reduce the Fe(III) oxides (Reguera et al., 2005). The conductive pili of *G. sulfurreducens* bind the mineral particles and function as an electronic conduit between the cell and the minerals during respiration (Reguera et al., 2005). Approximately one third of the reduced Fe(III) in the oxides is solubilized as Fe(II), while the rest remains in mineral form as magnetite particles attached to the pilus fibers (Reguera et al., 2005). The pili also bind the soluble uranyl ion and reduce it to a mononuclear uranium mineral phase, which remains attach to the pili (Cologgi et al., 2011). The attachment of magnetite or reduced uranium mineral particles to the pili prevents the conductive appendages from binding more electron acceptor and could halt respiration. However, PilT-mediated depolymerization of the pilins could allow the cells to retract the T4P and detach the reduced minerals. The pilins could then be recycled in a new round of polymerization to produce “clean” T4P, which are now ready to bind the electron acceptor and discharge metabolic electrons. In this manner, the antagonistic action of PilB and PilT could confer on *Geobacter* bacteria a competitive advantage for metal respiration. This would be particularly relevant in the natural environments inhabited by *Geobacter* bacteria, where Fe(III) oxides are often found dispersed in the extracellular medium. The dynamic nature of the T4P could also be critical for the cells to survive in uranium-contaminated environments, as rapid cycles of extension and retraction would promote the binding and reductive precipitation of more soluble uranyl cation, preventing its permeation and mineralization inside the cell envelope and preserving the cell's respiratory functions and viability (Cologgi et al., 2011).

It is important to note that our studies did not rule out the possibility that PilT3, one of the PilT paralogs in *G. sulfurreducens*, may be required to fine-tune the retraction of T4P under specific conditions. In *P. aeruginosa*, the PilT paralog PilU is recruited to the T4P apparatus to work coordinately with PilT to power twitching motility (Whitchurch and Mattick, 1994). As a result, mutations in either *pilT* or *pilU* lead to hyperpiliation and non-twitching phenotypes (Whitchurch and Mattick, 1994). While the *pilT* mutant of *P. aeruginosa* forms denser biofilms under static conditions, the *pilU* mutant is delayed in biofilm formation only under flow conditions (Chiang and Burrows, 2003). The delayed biofilm-forming abilities of the *pilU* mutant are similar to the delayed colonization of the anode electrode observed in MECs driven by the *pilT3* mutant of *G. sulfurreducens* (Figure 5B). In MECs, the anode medium is continuously stirred to promote the initial attachment of planktonic cells to the electrode and the diffusion of the electron donor and any other nutrients needed to support the growth of the anode biofilm (Speers and Reguera, 2012). The flow conditions that prevail in the MEC environment are likely to unmask defects from mutants with reduced adherence such as the *pilT3* mutant, similarly to how the phenotype of the *pilU* mutant of *P. aeruginosa* was unmasked in biofilm assays under flow (Chiang and Burrows, 2003). The reduced adherence of the *pilU* mutant of *P. aeruginosa* has been proposed to result from differences in the strength and/or integrity of the T4P (Chiang and Burrows, 2003). However, it is unlikely that T4P integrity was compromised in the *pilT3* mutant of *G. sulfurreducens* because pili-dependent functions such as the structure and electroactivity of anode biofilms was not affected (Figure 5) nor were the rates of Fe(III) oxide reduction affected (Figure 6A). Based on the cross-regulation of EPS production and pili synthesis that may exist in *G. sulfurreducens* (Steidl et al., 2016), it is possible that the *pilT3* mutation negatively influenced the synthesis of the Xap EPS, which *Geobacter* cells use to attach to positively-charged surfaces such as an anode electrode poised at a positive voltage, as in our MEC studies, or to Fe(III) oxides (Rollefson et al., 2011). The defect may have been transient, because once the cells attached, they grew as biofilms with the same structure and electrochemical activity as the WT (Figures 5B,C). Similarly, the *pilT3* mutant had an initial delay during the reduction of Fe(III) oxides (Figure 6A), which also depends on the ability of the cells to produce the EPS to anchor outer membrane *c*-cytochromes needed for electron transfer to the iron minerals (Rollefson et al., 2011). The coordinated regulation of the assembly of conductive T4P and synthesis of EPS could be analogous to *M. xanthus*, which uses EPS to trigger the retraction of the pili and relies on the pili dynamics to send feedback information to the Dif chemotaxis pathway to modulate EPS production (Black et al., 2006).

Alternatively, PilT3 may function as an ATPase in a separate apparatus such as a type II secretion system, which also relies on the ATPase activities of PilB- and PilT-like motors to polymerize and depolymerize pseudopilins, respectively, and export proteins across the outer membrane (Vignon et al., 2003). Indeed, *pilT3* was located downstream of a *pilB*-like gene (*mshE*) and the PilT3 and MshE proteins are highly expressed in Fe(III) oxide cultures of *G. sulfurreducens* (Ding et al., 2008), suggesting a conserved, coordinated function. PilT-like proteins can also be components of more evolutionary divergent secretion systems such as type IV secretion systems (T4SS), which translocate DNA or proteins across the cell envelope (Sexton et al., 2004). In these systems, PilT-like proteins function as traffic, rather than motor, ATPases, energizing the early steps of biogenesis of the T4SS or the translocation of the substrate (Alvarez-Martinez and Christie, 2009). PilT1, which in *G. sulfurreducens* is encoded by a gene in the *recA* operon (Figure 2B), is a likely candidate to function as a traffic ATPase in a T4SS system implicated in DNA uptake, because its expression would be coordinated with the recombination machinery. The function of PilT2, which is encoded by the orphan *pilT*2 paralog, is more difficult to predict. However, the fact that it is not conserved in other *Geobacter* spp. points at a role specific to the physiology of *G. sulfurreducens*.

In summary, we present genetic evidence that identifies PilT4 as the elusive PilT ATPase of the T4P apparatus of *G. sulfurreducens* and reveals a role for PilT-mediated pilus retraction during cellular respiration, both in electroactive biofilms and during the reduction of Fe(III) oxides. Whether PilT4 also requires the coordinated action of any of the other PilT paralogs, particularly PilT3, remains to be elucidated. Indeed, PilT3 complemented all the PilT functions assayed in *P. aeruginosa* and showed reduced adherence phenotypes during the colonization of electrodes and reduction of Fe(III) oxides previously linked to retraction defects in other bacteria (Chiang and Burrows, 2003). We cannot rule out that the conductive pili mediate twitching motility in *G. sulfurreducens* either. Both PilT3 and PilT4 rescued the non-twitching phenotype of a PilT-deficient mutant of *P. aeruginosa* (Figure 4D). Moreover, the inactivation of *pilT4* in *G. sulfurreducens* produced dense biofilms (Figure 5), a phenotype that could also result from defects in twitching motility (Chiang and Burrows, 2003). Twitching motility was not observed in *G. sulfurreducens* in submerged assays that used glass coverslips with or without iron coatings (Reguera et al., 2005). Such Fe(III) oxide coatings can be used as sole electron acceptor for respiration (Reguera et al., 2005, 2007) but may not be sufficiently smooth or uniform to promote cell translocation. Hence, assay modifications may be required to better mimic the surfaces and conditions that *Geobacter* bacteria encounter in the environment and which may select for twitching motility phenotypes. Lastly, direct visualization of pilus retraction could provide valuable insights into the mechanical properties of *Geobacter* pili that allow them to function as retractile appendages while functioning as electronic conduits during respiration.

## Author contributions

AS, BS, JH, and GR designed the experiments and analyzed the data. AS carried out the heterologous expression experiments, with assistance from AG, and phenotypically characterized the *Geobacter* mutants in microbial electrolysis cells and cultures with Fe(III) oxides. BS constructed the *Pseudomonas* strains used in the heterologous expression experiments and, with JH, the *Geobacter* mutant strains; BS also performed the biofilm assays in *G. sulfurreducens* and JH, the qRT-PCR analyses; together with GR they did comparative genomic and protein analyses. AS, BS, and GR co-wrote the paper. All authors discussed the results and commented on the manuscript.

### Conflict of interest statement

The authors declare that the research was conducted in the absence of any commercial or financial relationships that could be construed as a potential conflict of interest.
